# Telomere-to-telomere pear (*Pyrus pyrifolia*) reference genome reveals segmental and whole genome duplication driving genome evolution

**DOI:** 10.1093/hr/uhad201

**Published:** 2023-10-12

**Authors:** Manyi Sun, Chenjie Yao, Qun Shu, Yingyun He, Guosong Chen, Guangyan Yang, Shaozhuo Xu, Yueyuan Liu, Zhaolong Xue, Jun Wu

**Affiliations:** College of Horticulture, State Key Laboratory of Crop Genetics & Germplasm Enhancement and Utilization, Nanjing Agricultural University, Nanjing, Jiangsu 210095, China; Zhongshan Biological Breeding Laboratory, No.50 Zhongling Street, Nanjing, Jiangsu 210014, China; College of Horticulture, State Key Laboratory of Crop Genetics & Germplasm Enhancement and Utilization, Nanjing Agricultural University, Nanjing, Jiangsu 210095, China; Zhongshan Biological Breeding Laboratory, No.50 Zhongling Street, Nanjing, Jiangsu 210014, China; Institute of Horticulture, Yunnan Academy of Agricultural Sciences, Kunming 650205, China; Institute of Horticulture, Yunnan Academy of Agricultural Sciences, Kunming 650205, China; College of Horticulture, State Key Laboratory of Crop Genetics & Germplasm Enhancement and Utilization, Nanjing Agricultural University, Nanjing, Jiangsu 210095, China; Zhongshan Biological Breeding Laboratory, No.50 Zhongling Street, Nanjing, Jiangsu 210014, China; College of Horticulture, State Key Laboratory of Crop Genetics & Germplasm Enhancement and Utilization, Nanjing Agricultural University, Nanjing, Jiangsu 210095, China; Zhongshan Biological Breeding Laboratory, No.50 Zhongling Street, Nanjing, Jiangsu 210014, China; College of Horticulture, State Key Laboratory of Crop Genetics & Germplasm Enhancement and Utilization, Nanjing Agricultural University, Nanjing, Jiangsu 210095, China; Zhongshan Biological Breeding Laboratory, No.50 Zhongling Street, Nanjing, Jiangsu 210014, China; College of Horticulture, State Key Laboratory of Crop Genetics & Germplasm Enhancement and Utilization, Nanjing Agricultural University, Nanjing, Jiangsu 210095, China; Zhongshan Biological Breeding Laboratory, No.50 Zhongling Street, Nanjing, Jiangsu 210014, China; College of Horticulture, State Key Laboratory of Crop Genetics & Germplasm Enhancement and Utilization, Nanjing Agricultural University, Nanjing, Jiangsu 210095, China; Zhongshan Biological Breeding Laboratory, No.50 Zhongling Street, Nanjing, Jiangsu 210014, China; College of Horticulture, State Key Laboratory of Crop Genetics & Germplasm Enhancement and Utilization, Nanjing Agricultural University, Nanjing, Jiangsu 210095, China; Zhongshan Biological Breeding Laboratory, No.50 Zhongling Street, Nanjing, Jiangsu 210014, China

## Abstract

Previously released pear genomes contain a plethora of gaps and unanchored genetic regions. Here, we report a telomere-to-telomere (T2T) gap-free genome for the red-skinned pear, ‘Yunhong No. 1’ (YH1; *Pyrus pyrifolia*), which is mainly cultivated in Yunnan Province (southwest China), the pear’s primary region of origin. The YH1 genome is 501.20 Mb long with a contig N50 length of 29.26 Mb. All 17 chromosomes were assembled to the T2T level with 34 characterized telomeres. The 17 centromeres were predicted and mainly consist of centromeric-specific monomers (CEN198) and long terminal repeat (LTR) *Gypsy* elements (≥74.73%). By filling all unclosed gaps, the integrity of YH1 is markedly improved over previous *P. pyrifolia* genomes (‘Cuiguan’ and ‘Nijisseiki’). A total of 1531 segmental duplication (SD) driven duplicated genes were identified and enriched in stress response pathways. Intrachromosomal SDs drove the expansion of disease resistance genes, suggesting the potential of SDs in adaptive pear evolution. A large proportion of duplicated gene pairs exhibit dosage effects or sub-/neo-functionalization, which may affect agronomic traits like stone cell content, sugar content, and fruit skin russet. Furthermore, as core regulators of anthocyanin biosynthesis, we found that *MYB10* and *MYB114* underwent various gene duplication events. Multiple copies of *MYB10* and *MYB114* displayed obvious dosage effects, indicating role differentiation in the formation of red-skinned pear fruit. In summary, the T2T gap-free pear genome provides invaluable resources for genome evolution and functional genomics.

## Introduction

Telomere-to-telomere (T2T) genomes provide fully complete gapless genome assemblies of extremely high quality, with coherence in gene, centromeric, telomeric, and repetitive regions. A T2T genome is important for the deepest understanding of genome evolution and for best facilitating crop improvement. With advancements in long-read sequencing technologies, a number of T2T genomes have been assembled using Pacific Biosciences (PacBio) HiFi read, Oxford Nanopore Technology (ONT) ultra-long read, and high-throughput chromosome conformation capture (Hi-C) data. Recently, the first complete human T2T genome was assembled. It captured an additional 200 Mb of sequence data containing 1956 gene predictions (nearly 100 of which are predicted to encode proteins) [1]. Many T2T plant genomes have also been assembled, such as *Arabidopsis* [2], rice [3], maize [4], strawberry [5], watermelon [6], kiwifruit [7], and banana [8]. These genomes accurately represent high-complexity sequences in telomeric, centromeric, and high repeat regions, and provide an opportunity to explore genetic variations, repetitive sequences, and duplication events in these formerly ‘dark matter’ regions.

Pear is a wide-spread member of the Rosaceae family with a long history of cultivation, and it consisted of more than 22 species, as well as more than 5000 accessions with different morphological, physiological, and adaptive characteristics [9]. A recent report estimated annual worldwide pear production at ~18.99 million tons (2021, Food and Agriculture Organization of the United Nations). That report divided pears into two groups, namely Asian and European pears, with cultivars mainly consisting of five species: *Pyrus communis*, which is overwhelmingly cultivated in Europe; and *Pyrus pyrifolia*, *Pyrus bretschneideri*, *Pyrus ussuriensis*, and *Pyrus sinkiangensis*, which are commonly cultivated in Asia [10]. Eight pear genome assemblies were released to GDR (Genomic Database for Rosaceae) and NCBI. These genomes have promoted the development of functional genomics and further guide pear breeding. However, many gaps still exist in the genomes due to technology limitations, which results in a loss of genetic information and restricts our understanding of pear genome structure and evolution.

In most eukaryotic genomes, segmental duplication (SD) and whole genome duplication (WGD) are two major mechanisms that result in gene duplication [11, 12]. A duplicated gene may lose its function as a result of redundancy and end up being removed from the genome by natural selection. However, several duplicated genes are retained as a result of subfunctionalization or neofunctionalization (sub-/neo-functionalization), which provides a source of new genes. These novel genes may affect several agronomic traits, and can be used for genetic breeding. In a distantly-related wild citrus (*Atalantia buxifolia*), the *AgRuby2-AgRuby1* gene cluster, which encodes an anthocyanin activator, shows a pattern of subfunctionalization [13]. *AgRuby1* has a higher expression level than *AgRuby2* in pigmented leaves, but *AgRuby1* has a lower expression level than *AgRuby2* in mature fruit. These opposing expression patterns suggest different roles for anthocyanin accumulation in specific tissues. Gene duplication also generated the paralogs *ScAN1* and *ScAN2*, which show obvious subfunctionalization in potato (*Solanum* sp.) [14]. *ScAN1* is specialized for anthocyanin production, but in cold-tolerant potato species, expression of *ScAN2* can be induced by cold stress. Incomplete genomes may fail to capture such fine-scale genetic information, especially when it resides within duplicated regions, limiting our understanding of gene duplication and any subsequent sub-/neo-functionalization.

**Figure 1:**
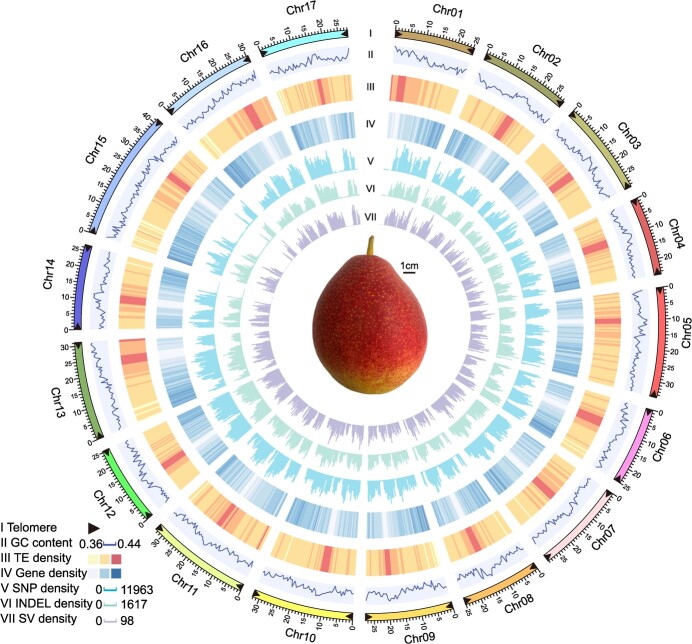
The telomere-to-telomere genome assembly of *Pyrus pyrifolia* ‘Yunhong No. 1’ (YH1). Genomic features of the YH1 genome and a mature fruit are displayed. I, distribution for the 34 telomeres; II, GC content density; III, TE density; IV, gene density; V, SNP density; VI: INDEL density; VII: SV density.

In this study, we generated the first pear T2T gap-free genome, which has provided the first opportunity for analysis of telomeric and centromeric regions. Furthermore, we used our T2T genome to identify SD regions, and found many SD events occurred both within and between chromosomes, resulting in gene duplications specialized for distinct stress responses. We investigated the divergence of duplicated genes using data from the T2T genome, transcriptome, and whole-genome bisulfite sequencing (WGBS). These duplicated genes may affect agronomic traits by dosage effects or sub-/neo-functionalization. Insights gained from these data will improve our understanding of the structure and gene function of the pear genome.

## Results

### A T2T gap-free reference genome for Yunhong no. 1

‘Yunhong No. 1’ (YH1), belonging to *P. pyrifolia*, originated in Yunnan in southwest China. It is a representative of red-skinned Asian pears, with two-thirds red skin coloration (Fig. 1). To generate the telomere-to-telomere genome, we incorporated multiple sequencing technologies including Illumina, PacBio HiFi, ONT ultra-long, and Hi-C. A total of 21.00 Gb HiFi reads using the PacBio Sequel IIe platform, and 74.87 Gb ONT-ultra long reads were generated. Hifiasm [15] was used for HiFi genome assembly using PacBio HiFi reads. The assembly contig N50 was 29.26 Mb. The ONT genome sequences were assembled using NextDenovo, and Illumina reads were used to polish the generated contigs. The contigs of the HiFi genome assembly and the ONT genome assembly were both anchored in 17 chromosomes using ALLHiC [16]. The ONT genome was then merged to the HiFi-assembled reference for filling the gaps. Juicer (v1.6) [17] was used to generate interaction maps using Hi-C data, and the orientation of all chromosomes was confirmed (Fig. S1A, see online supplementary material). The gap-free genome was generated with a genome size of 501.20 Mb (Table 1) (98.92% of estimated genome size, Fig. S1B, see online supplementary material). Using the plant-specific seven-base telomere repeat sequence (3′-TTTAGGG/5′-CCCTAAA) as a query [7], we identified all 34 telomeres (Fig. 1) and 17 gap-free T2T pseudomolecules of the YH1 pear genome.

Plant centromeres are essential for DNA division, but they are largely underexplored as a result of their complexity and high sequence repetition [18]. In this study, a centromeric-specific monomer with 198 bp length (CEN198) was predicted using Tandem Repeat Finder (TRF) and the cd-hit pipeline (see the Materials and methods section) [7]. Monomer locations were identified using the nhmmer search algorithm. The centromere boundaries were determined by combining the Hi-C interaction map, repetitive sequence, and gene density. Finally, 17 centromeric regions were predicted with sizes ranging from to 1.35 to 2.80 Mb (Fig. S2 and Table S1, see online supplementary material). The centromeric regions have high transposable element (TE) densities and low gene densities. CEN198 has high density at centromeric regions, but low density at other regions. TEs in centromeric regions mainly consisted of LTR of *Gypsy* elements with percentages ranging from 74.73% to 90.10%, which is considerably higher than what is seen at the level of the entire genome (Table S1, see online supplementary material). A total of 95 genes were identified from the centromeric regions, and 44 genes (46.32%) were expressed with a transcripts per million (TPM) value >1, which is lower than the percentage of expressed genes at the whole genome level (68.58%).

The quality and completeness of the YH1 genome were evaluated using multiple methods. First, the Illumina short reads were mapped to the genome. A 99.97% genome coverage suggested the high completeness of YH1 genome (Table 1). We used BUSCO to further evaluate genome completeness; it reported that 99.00% core genes (1598 out of 1614 BUSCOs) were complete. The calculated QV (assembly consensus quality value) using Merqury was 49.21, suggesting that the genome base call accuracy was higher than 99.99%. Moreover, long terminal repeat (LTR) annotation allowed the LTR assembly index (LAI) to be computed. Its value was 23.78, which, being above 20, meets the accepted standard for gold quality [19].

For genome annotation, we first screened, annotated, and mask repeated sequences in the genome. A total of 251.97 Mb of repeated sequences accounts for 50.27% of the YH1 genome, a percentage similar to that of the ‘Dangshansuli’ pear [20]. A total of 41 969 genes were annotated. They had a mean coding sequence length of 1097.81 bp, a mean number of exons of 4.74, and a mean exon length of 231.39 bp. These values are similar to those found for other sequenced Rosaceae species (Table S2, see online supplementary material). In total, 97.26% of the genes were functionally annotated by the Swissprot, NR, KEGG, InterPro, GO, or Pfam databases (Table S3, see online supplementary material). The annotated gene set captured 98.00% of the BUSCO 1614 reference gene set, which is higher than for previously released pear genomes [20–26] (Fig. S3, see online supplementary material). These results suggest the high quality and completeness of the YH1 gene set.

### Global comparison between YH1 T2T genome and two previously released *Pyrus pyrifolia* genomes

**Figure 2:**
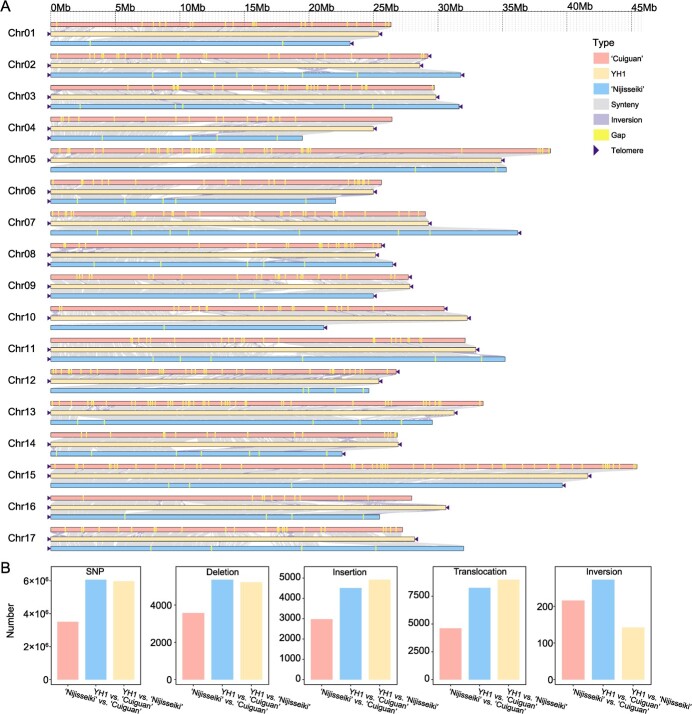
Collinearity and variation analysis of YH1 and two other *Pyrus pyrifolia* genomes (‘Cuiguan’ and ‘Nijisseiki’). (**A**) Collinearity analysis between three *P. pyrifolia* genomes. The YH1 genome was set as reference. (**B**) Histograms showing the number of SNPs, deletions, insertions, translocations, and inversions between each pair of genomes (YH1 vs. ‘Cuiguan’, YH1 vs. ‘Nijisseiki’, and ‘Nijisseiki’ vs. ‘Cuiguan’).

Compared with the previously released *P. pyrifolia* genomes (‘Cuiguan’ and ‘Nijisseiki’), the YH1 genome has significantly improved integrity, continuity, and accuracy (Fig. 2A). The N50 contig size of YH1 (29.26 Mb) is much higher than that of the ‘Cuiguan’ (1.28 Mb) and ‘Nijisseiki’ (7.60 Mb) genomes (Fig. S4A, see online supplementary material). However, the major improvement is in the absence of gaps, 442 of which were found in ‘Cuiguan’ and 76 in ‘Nijisseiki’ (Fig. 2A; Fig. S4B, see online supplementary material). In YH1, 34 telomeres were assembled on the 17 *P. pyrifolia* chromosomes, whereas only 7 and 18 telomeres were captured in the sequenced genomes of ‘Cuiguan’ and ‘Nijisseiki’, respectively (Fig. S4C, see online supplementary material). The BUSCO values showed that YH1 has higher completeness of gene structure annotation (98.00%) than ‘Cuiguan’ (95.97%) and ‘Nijisseiki’ (96.72%) (Fig. S2, see online supplementary material). These results suggest that the YH1 T2T genome is a higher quality *P. pyrifolia* reference genome.

**Table 1:** Summary of ‘Yunhong No. 1’ (YH1) genome assembly.

Genomic feature	YH1
Estimated genome size	506.65
Total size of assembled contigs (Mb)	501.20
Number of contigs	20
N50 value of contig length (Mb)	29.26
Anchor ratio (%)	99.81
Number of gap-free chromosomes	17
Number of telomeres	34
Number of predicted centromeres	17
Percent of repeat sequence (%)	50.20
Genome BUSCOs (%)	99.00
LTR assembly index score	23.78
Number of genes/transcripts	41 969
Gene BUSCOs (%)	98.00
QV value	49.21
Mapping rate (%)	98.97
Coverage (%)	99.97

We also identified the variations between three *P. pyrifolia* genomes, and a quantity of SNPs and structural variations (SVs, including insertions, deletions, translocations, and inversions) were identified (Fig. 2B). Many more variations were identified between YH1 and ‘Cuiguan’ or ‘Nijisseiki’ than between ‘Cuiguan’ and ‘Nijisseiki’. A total of 6.07 [5.97] Mb of SNPs were identified between YH1 and ‘Cuiguan’ [‘Nijisseiki’] which was about 1.73-fold [1.70-fold] of the SNPs between ‘Cuiguan’ and ‘Nijisseiki’ (3.51 Mb). Meanwhile, the number of deletions between YH1 and ‘Cuiguan’ [‘Nijisseiki’] was approximately1.50-fold [1.46-fold] greater than that between ‘Cuiguan’ and ‘Nijisseiki’. The number of insertions between YH1 and ‘Cuiguan’ [‘Nijisseiki’] was about 1.51-fold [1.65-fold] greater than that between ‘Cuiguan’ and ‘Nijisseiki’. The number of translocations between YH1 and ‘Cuiguan’ [‘Nijisseiki’] was 1.79-fold [1.95-fold] of that between ‘Cuiguan’ and ‘Nijisseiki’. However, the number of inversions between YH1 and ‘Nijisseiki’ was lower than that between YH1 and ‘Cuiguan’ and also between ‘Cuiguan’ and ‘Nijisseiki’. These variations might change the gene structure and may alter regulation regions, potentially resulting in the phenotypic variance between pear accessions.

### Segmental duplications contributing to pear genome evolution

**Figure 3:**
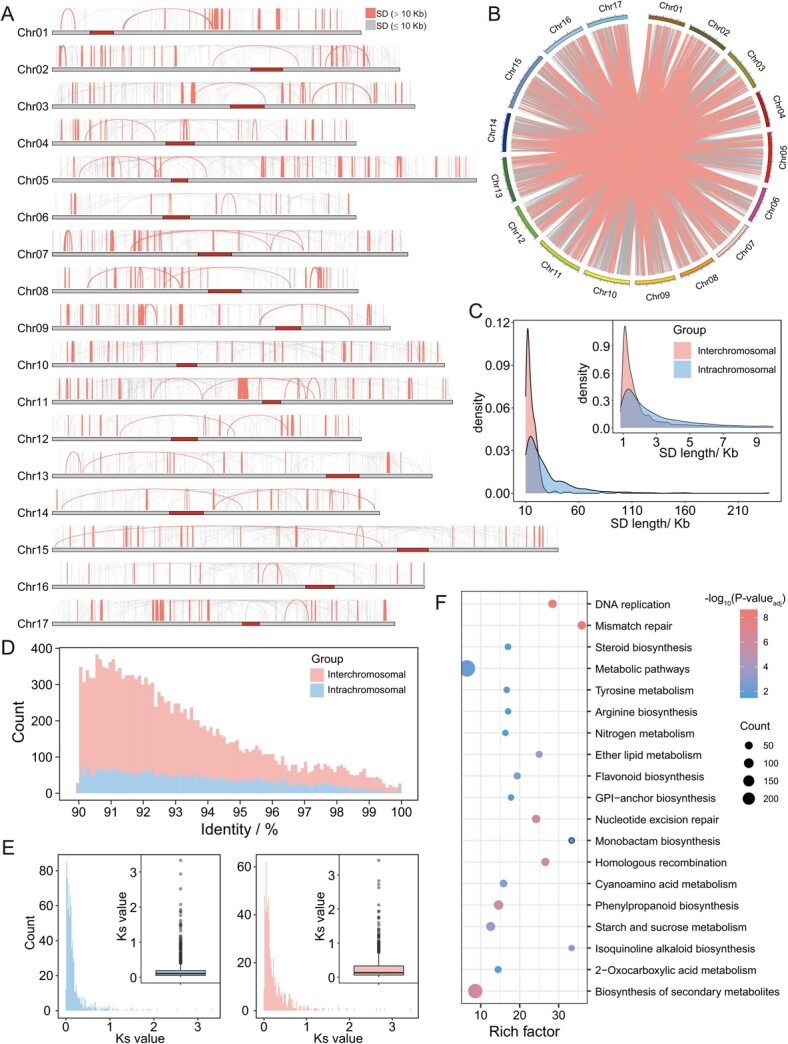
Segmental duplication (SD) analysis in the YH1 genome. (**A**) Distribution of intrachromosomal segmental duplication. (**B**) Distribution of interchromosomal segmental duplication. . (**C**) Density plot of SD length distribution. (**D**) Histogram comparing sequence identity of interchromosomal SDs and intrachromosomal SDs. (**E**) Histogram and boxplot showing the Ks value distribution of genes in intrachromosomal (left) and interchromosomal SDs (right). (**F**) KEGG enrichment results of genes in SD regions (adjusted *P*-value <0.05).

SDs are repeated DNA sequences longer than 1 kb with at least 90% nucleotide identity within the genome [27]. SDs are hotspots of genome instability and can result in gene copy number variance and functional innovation [28, 29]. Due to assembly technology limitations and the complexity of SDs, SD regions are often incorrectly assembled, collapsed (mistakenly aligned to the same region), or lost, which reduces our understanding of the evolution of the pear genome. These SD regions account for 10.76% of the YH1 genome sequence (53.94 Mb / 501.20 Mb) (Fig. 3A and B); 6.27% SDs (1035 / 16 504) were larger than 10 kb. In YH1, SDs occurred at higher frequencies on chromosome 11 (Chr11), Chr17, and Chr04 (Table S4, see online supplementary material), and at lower frequencies on Chr16, Chr13, and Chr12, suggesting SDs were not equally distributed on each chromosome.

A total of 78.14% (12 896 / 16 504) of the SDs were identified as having occurred between chromosomes, which was greater than the percentage of SDs (21.86%, 3608 / 16 504) that occurred within chromosomes. The average length of intrachromosomal SDs (7732.52 bp) was higher than that of interchromosomal SDs (2307.69 bp) (Fig. 3C). Intrachromosomal SDs showed higher sequence identity than interchromosomal SDs (Fig. 3D). The SD-driven duplicated genes were identified with at least 50% of the full-length gene maps to an SD region, and 1531 pairs were identified in SD regions. We also calculated the Ks values of these duplicated genes as proxies for the generation time of their corresponding SDs. We found that the Ks values of 58.85% (901 / 1531) of the duplicated gene pairs were lower than 0.15, suggesting that these gene duplications occurred after a recent WGD event [30]. The Ks values of duplicated genes in interchromosomal SDs (average Ks = 0.26) was significantly higher (*P*-value = 1.96e-07, Wilcoxon rank sum test) than that of duplicated genes in intrachromosomal SDs (average Ks = 0.20) (Fig. 3E). Thus, SD is seen to play an important role in gene duplication and pear genome evolution.

SD-driven duplicated genes can result in plant phenotype variance, which can increase environmental adaptation [31, 32]. KEGG pathway enrichment analysis showed that the SD-duplicated genes were mainly enriched in metabolic pathways (Fig. 3F), including steroid, flavonoid, phenylpropanoid biosynthesis, and tyrosine metabolism, which may contribute to stress responses [33–36]. Furthermore, 18 disease resistance gene pairs were exclusively identified in intrachromosomal SDs (Table S5, see online supplementary material) and all these SDs were intrachromosomal SDs, suggesting that intrachromosomal SDs may participate in the expansion of disease-resistance genes. In addition, we identified an enrichment in the starch and sucrose metabolism pathway. SD-driven copy number increases of sucrose synthase, starch synthase, and hexokinase may affect the sugar content of fruit flesh [37] (Table S6, see online supplementary material).

### Expression divergence after gene duplication

**Figure 4:**
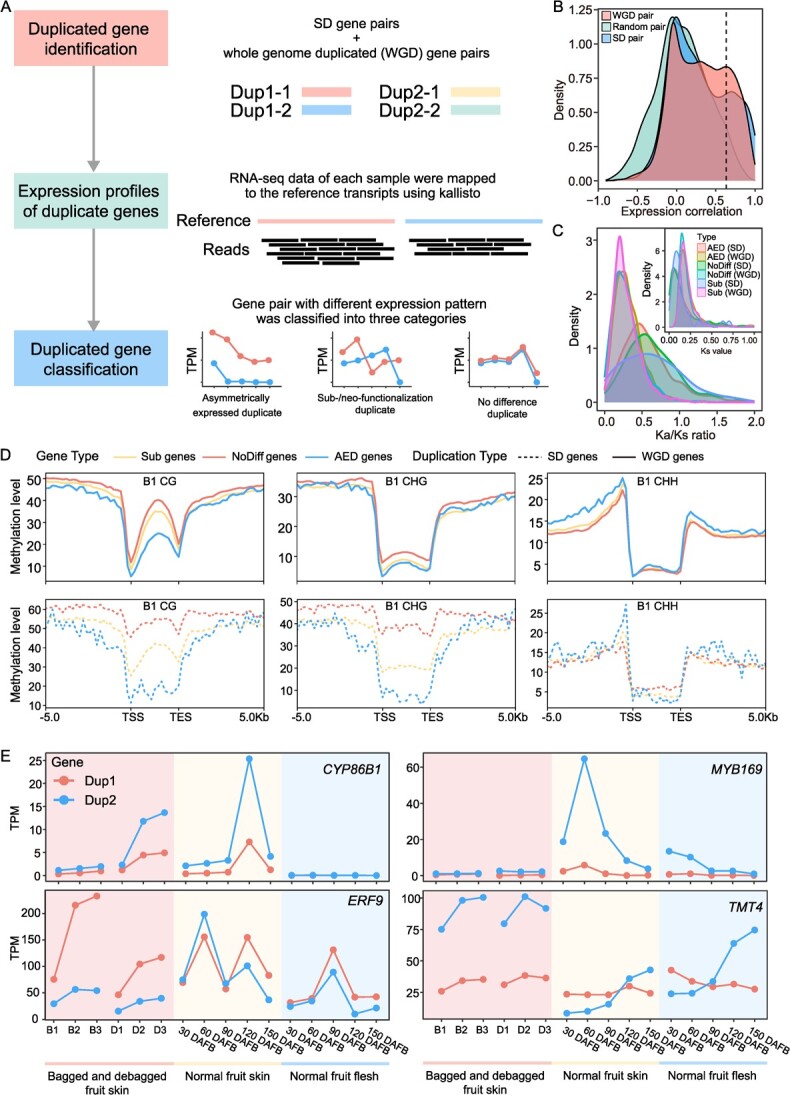
Analysis of pear gene duplication and divergence. (**A**) Distribution of asymmetrically expressed duplicate (AED), sub-/neo-functionalization (Sub), and no difference (NoDiff) gene pairs. Detailed information can be found in the Materials and methods section. (**B**) Density plots of the Pearson correlation coefficient between gene pairs in instances of segmental duplication (SD), whole genome duplication (WGD), and random gene pairs (see below). All duplicated gene pairs identified from Whole-Genome Duplication Integrated analysis (WGDI) were classified as WGD gene pairs. 10 000 gene pairs were randomly selected using the ‘random’ module in Python. (**C**) Distribution of Ks and Ka/Ks ratio of AED, Sub, and NoDifff gene pairs in SD and WGD gene pairs. (**D**) CG, CHG, and CHH methylation level of AED, Sub, and NoDiff genes in the bagged fruit skins (B1) sample. Dotted lines represent SD genes and continuous lines represent WGD genes. (**E**) Expression pattern of four duplicated gene pairs (*CYP86B1*, *MYB169*, *ERF9*, and *TMT4*) in different fruit samples. Dup1 and Dup2 represent the two duplicated genes. D1, D2, and D3 represent debagged fruit skins at 4, 8, and 10 days after bag removal, respectively; B1, B2, and B3 represent bagged fruit skins at 4, 8, and 10 days after bagging, respectively; 30, 60, 90, 120, and 150 DAFB represent fruit at the specified number of days after flower bloom

Gene duplication mainly contributes to phenotypic change and adaptive evolution in plants by introducing new genes and driving function divergence [38, 39]. Although duplicated genes are functionally redundant and tend to form pseudogenes, some of them survive by dosage reduction or by sub-/neo-functionalization [40]. To reveal the divergence of duplicated genes in pear, we identified the duplicated genes and then used the transcriptome and WGBS data to assess the divergence of duplicated genes at the transcription and methylation levels. First, gene pairs that arose through SD or recent WGD events were identified (see Materials and methods) (Fig. 4A). Second, RNA-seq data were mapped to reference transcripts using kallisto [41]. Finally, based on the expression results in multiple samples, the duplicated genes were classified into the following three categories: asymmetrically expressed duplicate (AED: one constituent has a higher expression level in at least one third of the samples, and never has a lower expression level in the remaining samples), sub-/neo-functionalization (Sub: both genes of a pair have a higher expression level than the other in at least one sample), and no difference (NoDiff, duplicated genes could not be classified as AED or Sub) genes.

A total of 1531 SD and 12 256 WGD gene pairs were identified. Compared with random gene pairs, SD or WGD gene pairs have highly correlated expression levels (Fig. 4B). Of the 1531 SD gene pairs, 738 were identified as AED, 33 as Sub, and 760 as NoDiff gene pairs. Among the 12 256 WGD pairs, 6757 were identified as AED, 843 as Sub. and 4656 as NoDiff gene pairs. The Ks peak value for WGD gene pairs was about 0.15, which is larger than that of SD gene pairs. This suggests that a large fraction of the SD gene pairs arose after a recent WGD event. High proportions of SD genes have Ka/Ks values larger than one, suggesting that more SD genes are under positive selection pressure (Fig. 4C).

Changes in the level of methylation can regulate gene expression [42]. We quantified the methylation level (CG, CHG, and CHH) of duplicated genes, and found that different methylation patterns often occurred in the three gene categories (AED, Sub, and NoDiff) between SD and WGD gene pairs (Fig. 4D;Fig. S5, see online supplementary material). For both SD and WGD gene pairs, AED genes had lower methylation levels than Sub and NoDiff genes in the CG context. In the CHG context, the three categories from SD genes also showed obviously different methylation levels, but no difference was observed in WGD gene pairs. In the CHH context, no difference was observed between SD and WGD genes in any of the three categories. Additionally, 4214–6826 duplicated gene pairs showed significant correlation between methylation (CG, CHG, and CHH) and expression level (Table S7, see online supplementary material). These gene pairs were enriched in metabolic, secondary metabolites, fatty acid, amino acids, citrate cycle, and carbon metabolism KEGG pathways (Fig. S6, see online supplementary material). Different methylation patterns may be a major reason for the divergence of the three categories of duplicated genes, which may further affect the corresponding biological processes in pear.

**Figure 5:**
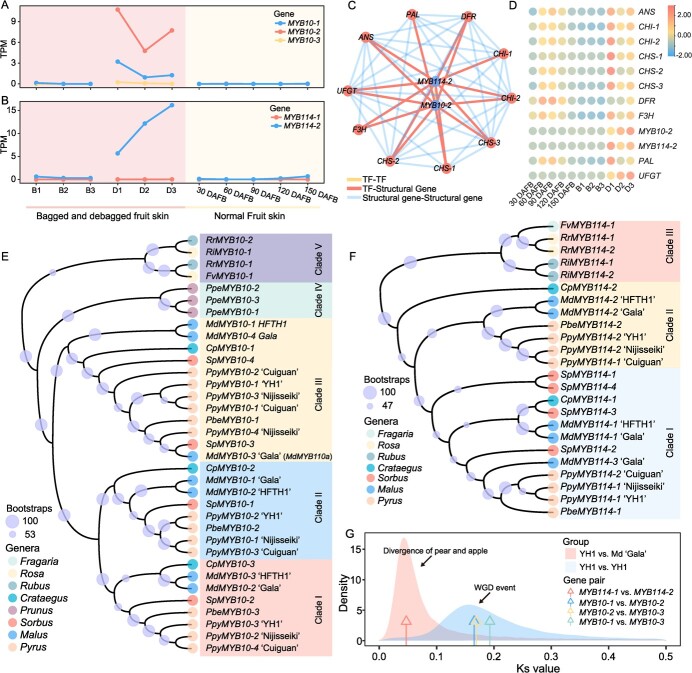
Duplication and dosage effect analysis of *MYB10* and *MYB114*. (**A**, **B**) The expression pattern of *MYB10* (**A**) and *MYB114* (**B**) duplicated genes in different fruit skin samples. ‘-1’, ‘-2’, and ‘-3’ represent three duplicated genes. D1, D2, and D3 represent debagged fruit skins at 4, 8, and 10 days after bag removal, respectively; B1, B2, and B3 represent bagged fruit skins after 4, 8, and 10 days of having been bagged, respectively; 30, 60, 90, 120, and 150 DAFB represent fruit at the corresponding number of days after flower bloom. (**C**) The co-expression network of *MYB10–2*, *MYB114–2*, and anthocyanin-related genes. (**D**) Heatmap showing the expression patterns of *MYB10–2*, *MYB114–2*, and anthocyanin-related genes. Red represents a high expression level. (**E**–**F**) The phylogenetic analysis of *MYB10* (**E**) and *MYB114* (**F**) duplicated genes in different genera. Ppy *Pyrus pyrifolia,* Pbe *P. betulifolia*, Ppe *Prunus persica*, Md *Malus domestica*, Cp *Crataegus pinnatifida*, Sp *Sorbus pohuashanensis*, Fv *Fragaria vesca*, Rr *Rosa rugosa*, and Ri *Rubus idaeus*. (**G**) Ks values of *MYB10* and *MYB114* duplicated gene pairs in the YH1 genome.

Many AED and Sub genes have been shown to be associated with fruit development and quality (Fig. 4E). *CYP86B1*, a member of the cytochrome P450 monooxygenase (CYP) family, participates in the formation of fruit skin russeting [43]. One of the two duplicated genes was more highly expressed in all samples than its partner, suggesting it was more important for russeting formation than its partner. *MYB169* can activate lignin biosynthesis and regulate secondary wall formation of fruit stone cells [44]. *MYB169* showed strong AED, in that one copy was highly expressed in early fruit developmental stages, which is consistent with the stone cell formation pattern. This result indicated that the *MYB169* duplicate may persist by having a reduced expression level, such that it no longer participates in pear stone cell formation. In addition, *ERF9*, which encodes an ethylene response factor, can inhibit anthocyanin biosynthesis in pear [45]. The expression levels of duplicated gene pairs suggested its sub-/neo-functionalization. *TMT4* encodes a tonoplast monosaccharide transporter, and is a major contributor to soluble sugar accumulation in pear fruit [46]. The duplicated gene pair of *TMT4* showed strong sub-/neo-functionalization. *TMT4* Dup2 was highly expressed in late fruit developmental stages, but the expression level of Dup1 decreases in late stages, demonstrating that Dup1 and Dup2 of *TMT4* played different roles in sugar accumulation during fruit development. These results showed that the divergence of duplicated genes can generate new desirable traits and provide genetic resources for pear breeding.

### Duplication and function divergence of *MYB10* and *MYB114*

*MYB10* and *MYB114* are two core transcription factors contributing to pear anthocyanin biosynthesis [47]. In this study, three copies of *MYB10* and two copies of *MYB114* were identified from the YH1 genome, and each pair showed a strongly asymmetric gene expression pattern (Fig. 5A). The *MYB10–2* gene was more highly expressed than the other two copies (*MYB10–1* and *MYB10–3*) in debagged fruit skin samples. In addition, only *MYB114–2* was expressed in debagged fruit skin samples. Low expression levels (TPM value lower than 0.2) of *MYB114–1* were observed in all samples (Fig. 5B). WGCNA analysis showed that *MYB10–2* and *MYB114–2* were co-expressed with anthocyanin biosynthesis*-*related genes like *CHS*, *DFR*, *ANS*, and *UFGT* (Fig. 5C and D), providing evidence that *MYB10–2* and *MYB114–2* are important regulators in anthocyanin biosynthesis. A transient transgene experiment showed anthocyanin accumulation was observed and the expression level of anthocyanin biosynthesis related genes was increased in pear fruit of overexpressed ‘*MYB114–2*’ and ‘*MYB10–2*’, which further validated the function of *MYB10–2* and *MYB114–2* (Fig. S7, see online supplementary material). In addition, low expressed copy (*MYB10–3* and *MYB114–1*) showed higher CG and CHG methylation level than high expressed copy (Fig. S8, see online supplementary material) indicating a role of methylation in the asymmetrical expression pattern of *MYB10* and *MYB114* [48]. These results indicated that only one copy of *MYB10* and *MYB114* was necessary for anthocyanin biosynthesis, and other copies may have reduced expression, and hence may not contribute to the production of anthocyanin in fruit skin.

We also collected genome sequences from the following nine prominent Rosaceae species: *Malus domestica*, *P. pyrifolia*, *Pyrus betuleafolia*, *Sorbus pohuashanensis*, *Crataegus pinnatifida*, *Prunus persica*, *Fragaria vesca*, *Rosa rugosa*, and *Rubus idaeus* (Table S8, see online supplementary material), and identified their orthologous *MYB10* and *MYB114* genes using *MYB114* (MF489219) [47] and *MYB10* (KT601121) [49] from pear and the *Arabidopsis thaliana* PAP1–PAP4 MYB TFs as queries. *MYB10* was identified in the genomes of *M. domestica*, *P. pyrifolia*, *P. betuleafolia*, *S. pohuashanensis*, *C. pinnatifida* and *Prunus persica* (Table S9, see online supplementary material). Three copies were identified in the *Prunus persica* genome, and three to four copies were identified in the genomes of *M. domestica, P. pyrifolia, P. betuleafolia, S. pohuashanensis*, and *C. pinnatifida*, which all underwent recent WGD events [30, 50]. Further phylogenetic analysis revealed that copies of MYB10 in *P. pyrifolia, P. betulifolia*, *M. domestica*, *C. pinnatifida* and *S. pohuashanensis* were distributed across three clades (Fig. 5E), but the three MYB10 in *Prunus persica* were clustered into another single clade. These results suggest that the *MYB10* duplication event in *Prunus persica* may not be common to other species which experienced the recent WGD events.

For *MYB114*, one to four copies were identified from the genomes of *M. domestica, P. pyrifolia, P. betuleafolia, S. pohuashanensis*, and *C. pinnatifida*, and all copies of *MYB114* from *Pyrus* species were clustered into two clades (Fig. 5F). The Ks values of three *MYB10* copies in pear ranged from 0.17 to 0.19, which overlaps with the peak Ks value of the most recent WGD event (Fig. 5G), indicating that the *MYB10* gene duplication may have taken place during that WGD event. The Ks values of two *MYB114* copies indicate that the *MYB114* duplication may have occurred during the time when pear and apple diverged. These results suggest that *MYB10* and *MYB114* underwent separate gene duplication events and that lowering gene expression may have helped to preserve the copies in pear.

## Discussion

An accurate and complete genome is helpful for breeding and crop genetic research. Since the first pear genome assembly was released in 2013 [20], several chromosome-level or contig-level pear genomes have been released [21–25, 51]. However, those genomes all contained gaps, as a result of their not including repetitive regions, such as centromeres and telomeres, which resulted in the loss of genetic information. In this study, we described the first T2T gap-free *de novo* genome assembly for pear (YH1) generated using HiFi, ONT ultra-long, and Hi-C data, resulting in higher quality and contiguity than is found in previously sequenced pear genomes. A total of 34 telomeres were assembled and identified, suggesting that all (17) chromosomes of the YH1 genome were assembled telomere-to-telomere. We successfully identified centromere-specific monomers and predicted 17 centromeric regions. All centromeric regions consisted of a high percentage of repetitive sequences, most of which were LTR *Gypsy* elements. This is consistent with other plant species like rice [3], rose [52], and maize [4]. The complete YH1 genome will provide opportunities for genome structure and functional gene analysis in pear.

With improvements in genome sequencing, SDs, which are a source of new genes and functions [53], have been analysed in human [54], non-human primates [55], mouse [56], rice [31], and barley [29]. In this study, 53.94 Mb of SD regions were identified, accounting for 10.76% of the sequenced genome, and suggesting their importance in genome structure and evolution [31]. This percentage is lower than that of the rice genome [31], but higher than that of the human genome [54]. SDs are one of the driving forces for variance of gene copy number and gene family expansion, and ultimately affect plant morphology and adaptation [31, 57, 58]. In this study, 1531 duplication pairs were identified in SD regions that are significantly enriched in stress response pathways. Furthermore, disease resistance genes were found exclusively in intrachromosomal SDs. These results suggested the role of SDs in enhancing the capacity for environmental adaptation in pear.

The Maleae-specific WGD event that occurred 30–45 MYA [20] resulted in a vast number of duplicated gene pairs in pear. Combined with SD-driven duplicated pairs, these duplicated genes experienced different fates (Fig. 3). A high percentage of duplicated genes show asymmetric expression patterns, suggesting that many duplicated genes are retained by reducing their expression levels to those of single-copy genes [38]. In addition, sub-/neo-functionalized genes may change crop phenotypes, like *AgRuby1* and *AgRuby2*, which regulate anthocyanin biosynthesis in different citrus tissues [13], *OsTb1* and *OsTb2*, which have opposite functions in rice tillering [59], and *GhERF1–7A*/*7D*, which exhibit functional divergence in cotton stress tolerance and yield [60]. In this work, 7495 gene pairs displayed asymmetrical expression patterns, and 876 duplicated gene pairs in the YH1 genome appear to have undergone sub-/neo-functionalization. Duplicated genes that are associated with important agronomic traits can serve as resources for pear genetic improvement.

Red fruit skin is now considered to be a crucial agronomic characteristic for commercial pears. Two R2R3-MYB transcription factors, *MYB10* and *MYB114*, are essential for anthocyanin biosynthesis in pear [47]. In this study, we confirmed that these two TFs are present in multiple copies in pear. *MYB10* duplication occurred during the period of the Maleae-specific WGD event. Presently, multiple copies of the *MYB10* gene show asymmetric expression patterns, implying that they were preserved by having reduced expression to achieve dosage balance [38]. Interestingly, *MdMYB10* presents sub-/neo-functionalization in apple [61]. *MdMYB10* is expressed and promotes anthocyanin biosynthesis in apple skin, flesh, and foliage. As a paralog of *MdMYB10, MdMYB110a* (*MdMYB10–3* ‘Gala’) is only expressed in the fruit cortex, late during development. These results demonstrate that genes duplicated from a single ancestor gene may have different destinies in different species.

In summary, our work represents the completion of a gap-free T2T pear genome replete with all 34 telomeres and 17 centromeres. We furthermore utilized it for analyses of genome duplication and divergence, and found that SDs play an important role in development and in the pear stress response, and that many duplicated genes have been retained by dosage balance or sub-/neo-functionalization. From this initial foray, one can already clearly conclude that our T2T genome and related genetic information facilitate trait dissection and allow for the genetic improvement of *P. pyrifolia*, the Asian cultivated pear.

## Materials and methods

### Sample collection

‘Yunhong No. 1’ (YH1) specimens were sampled at Anning experiment station of the Yunnan Academy of Agricultural Sciences, Yunnan Province, China. Young pear leaves were collected for DNA extraction. Additionally, young stem, mature stem, young leaf, mature leaf, as well as fruit at different developmental stages were all used for RNA extraction and RNA sequencing. All samples were quick-frozen with liquid nitrogen and stored in freezers (−80°C).

### PacBio, ultra-long ONT, Illumina, and Hi-C sequencing

DNA was extracted using a plant genomic DNA kit from TIANGEN, and its corresponding library was generated using a NEBNext Ultra II DNA Library Prep Kit for Illumina (Massachusetts, USA). The Illumina NovaSeq6000 platform was used to obtain 32 Gb of short read length sequencing data. For PacBio HiFi library construction, more than 5 μg of sheared DNA was subjected to size-selection on a BluePippin instrument (Massachusetts, USA). Approximately 20 kb PacBio Sequel IIe single-molecule real-time (SMRT) bell libraries were prepared according to the PacBio protocol. The library was loaded in SMRT Cells using DNA Sequencing Reagent Kit, and the SMRT cells were run on a PacBio RSII-CCS system, which generated 21 Gb of long-read data. The Ultra-long ONT sequencing library was prepared according to the Nanopore protocol. A total of 74.87 Gb ONT reads were generated with max extended read reaching 587.50 kb. The Hi-C library was constructed from young leaves by the Novogene Corporation Inc. (Beijing, China) using a previously described technique [62]. A total of 52.55 Gb of 150 bp paired-end reads were produced on the Illumina NovaSeq6000 platform.

### Transcriptome sequencing

To assist in gene prediction, RNA sequencing (RNA-seq) data was generated from samples from tender stem, mature stem, young leaf, seed, and fruit at different developmental stages (30, 60, 90, 120, and 150 days after flower bloom). Total RNA was extracted using the RNA Nano 6000 Assay Kit for the Bioanalyzer 2100 system, and RNA-Seq libraries were constructed according to the protocol provided by Illumina and sequenced on the NovaSeq6000 platform.

### Genome survey, assembly, gap filling, and assessment

Illumina reads were first used to estimate genome size and heterozygosity with Jellyfish [63] and GenomeScope [64] with 21-kmer. For genome assembly, Pacbio HiFi reads were *de novo* assembled to contigs using hifiasm [15] (v0.16.1-r375) with default parameters. Redundant sequences were removed using purge_dups (v1.2.5). Ultra-long ONT reads were filtered for N50 > 100 kb and predicted Q score of at least 7. The remaining ONT reads were *de novo* assembled to contigs using NextDenovo (v2.5.2, https://github.com/Nextomics/NextDenovo), and the resulting contigs were polished using NextPolish [65] with 50× Illumina data. Subsequently, the Hi-C data was used to correct and scaffold contigs using HiCUP [66] and ALLHiC [16], and purge_dups [67] (v1.2.5) were used to check and remove the redundancy of unanchored contigs with default parameters. Scaffolds were checked and refined using Juicebox [68] (v1.11.08). The genome assembled using PacBio HiFi data was selected as the reference genome, and the genome assembled using ONT data was merged to the reference genome for gap filling to obtain the final gap-free YH1 genome.

To evaluate the quality and completeness of the assembly, clean sequencing reads were mapped to each haplotype using BWA (v0.7.17). Then, SAMtools (v1.14) was used to calculated genome coverage and mapping rate. LAI (LTR Assembly Index) was calculated using LTR_retreiver. BUSCO [69] (1614 core plant conserved genes) (v5.22) and CEGMA [70] were used to evaluate genome completeness. The Qv (quality value) was calculated to evaluate whole genome base accuracy using Merqury [71] with default parameters.

### Identification of centromeres and telomeres

TRF (v4.09) [72] was used to identify the centromeric tandem repeat with parameters ‘2 7 7 80 10 50 2000 -f -d -m -l 15’. All Monomer elements were clustered using cd-hit (v4.8.1) [73] with parameters ‘-c 0.8 -T 70 -M 100000 -d 100’. nhmmer (v3.3.2) was then used to search for the locations of candidate centromeric repeats [74]. The nhmmer result, gene density, TE density, and Hi-C interaction map were used to determine the boundaries of each centromere. Telomeres were identified using tidk (https://github.com/tolkit/telomeric-identifier) and bowtie2 (v2.4.4) [75] with the plant-specific telomeric sequence ‘(3′-TTTAGGG/5′-CCCTAAA)n’ as the query.

### Repetitive sequence and gene annotation

Tandem repeats were identified using TRF with default parameters. The masked genome sequence was used for further TE identification. LTR_FINDER [76] and RepeatModeler (http://www.repeatmasker.org/RepeatModeler/) were used to build the *de novo* repeat sequence library. The *de novo* and known repeat libraries were then merged, and RepeatMasker (http://repeatmasker.org/) was used to annotate the ‘new’ repeat regions based on this merged library, and to deduce TE divergence.

The structure of protein-coding genes was predicted by combing two methods: *de novo* RNA-seq data and homology-based prediction. Protein sequence of *A. thaliana*, *Prunus persica*, *P. communis*, *P. betulifolia*, and *M. domestica* were downloaded. TBLASTN (v2.2.26) [77] and GeneWise (v2.4.1) [78] were used to predict the gene structure of the BLAST hits. Augustus (v3.4.0) [79], SNAP [80], and GlimmerHMM (v3.0.4) [81] were used for *de novo* gene prediction. Trinity was used for *de novo* RNA-seq transcript assembly, and the result was used for transcript annotation using PASA [82]. EVidenceModeler [83] (EVM) (v2.0.0) was used to integrate these prediction results into weighted consensus gene structures.

Gene functions were predicted by aligning the protein sequences to the Swiss-Prot (http://web.expasy.org/docs/swiss-prot/guideline.html) and NR database using BLAST search (with threshold E-value ≤1e–5). The motifs and domains were annotated using InterProScan [84] (v5.31) by searching against publicly available databases. The Gene Ontology (GO) IDs for each gene were extracted from the corresponding InterPro entry, and gene sets were mapped to KEGG (Kyoto Encyclopedia of Genes and Genomes) pathways for KEGG annotation. Transfer RNA genes were predicted using tRNAscan-SE (v1.3.1) [85]. Ribosomal RNA sequences of related species were selected as references for predicting rRNA sequences using BLAST [77]. INFERNAL (v1.1.2) [86] was used with its default parameters to identify miRNAs and snRNAs.

### Genome comparison and variation identification

Genome comparisons between YH1 and two other assemblies of *P. pyrifolia* (‘Cuiguan’ and ‘Nijisseiki’) were performed using nucmer (v4.0.0rc1) [87] with parameters ‘--mum -c 90 -l 40’. This produces a delta alignment file, which was processed using the delta-filter utility with the option ‘-1’ to obtain a ‘1-to-1’ alignment with each of the other two assemblies. The results were fed to the SyRI [88] pipeline, which used them to identify syntenic blocks, structural variations (insertions, deletions, duplications, translocations, and inversions), and sequence divergence.

### Identification of SD regions and Ks calculation of duplicated gene pairs

Briefly, genome assembly of YH1 was soft-masked with all repetitive sequences converted to lowercase letters. Segmental duplications (SDs) were identified using SEDEF [89] with default parameters. Then, SD sequences with identity ≥90%, sequence length ≥1000 bp were retained following previous standards [27, 31, 54, 90]. Those SDs that did not occur in a collinear block (e.g., exclude WGDs) were selected for further analysis. SD gene pairs were identified at least 50% of the full-length gene maps to an SD region.

WGD gene pairs were identified using the WGDI command-line tool [91]. Collinear genes were identified with the parameter ‘-icl’, and collinear gene dot plots were used to display the blocks. The Ks values between collinear genes were estimated using the Nei–Gojobori approach. Based on the Ks values and collinear gene dot plots, candidate WGD blocks (with Ks values ranging from 0.15–0.30) [20] were identified. Finally, the WGD gene pairs in the blocks were extracted with parameter ‘-a’. The protein sequences of each duplicated gene pair were aligned using MAFFT (v7.49) [92] and were then preferentially aligned to predicted coding sequences using ParaAT (v2.0) [93]. We then calculated the numbers of non-synonymous substitutions per synonymous site (Ka), synonymous substitutions per synonymous site (Ks), and the Ka/Ks ratios based on the NG (Nei-Gojobori) Ka and Ks estimation method implemented in PAML (v4.9b) [94].

### Sub-/neo-functionalization analyses of duplicated genes in the pear genome

RNA-seq reads from YH1 tissue samples with three biological replicates were collected (debagged fruit skins at 4 (D1), 8 (D2), and 10 (D3) days after bag removal, and bagged fruit skins on corresponding days (B1, B2, and B3); fruit flesh and skin collected at 30, 60, 90, 120, and 150 days after flower bloom). RNA-seq reads were trimmed using Trimmomatic (v0.39) [95]. Thereafter, kallisto [41] was used for TPM (fragments per kilobase of transcript per million mapped reads) estimation. Differential gene expression (DEG) analyses between duplicate gene pairs for each tissue were performed using DESeq2 with an FDR (false discovery rate) cut-off of 0.05 and |log2 fold change| cut-off of 1. Duplicated gene pairs were classified into three categories [38]: (i) sub-/neo-functionalized pairs (Sub): each duplicate was more highly expressed than the other in at least one sample; (ii) asymmetrically expressed duplicate (AED): one duplicate was more highly expressed in at least one third of the samples, and its expression was not lower than that of its partner in any samples; and (iii) the remaining duplicates were classified as no-difference (NoDiff) pairs.

The WGBS (whole-genome bisulfite sequencing) data from YH1 tissue samples with D1, D2, D3, B1, B2, and B3 were collected from a previous study [96]. The WGBS reads were filtered using Trim_Galore (v0.6.10) with default parameters (https://github.com/FelixKrueger/TrimGalore). The reference genome was indexed using the bismark_genome_preparation tool from Bismark (v0.24) [97]. Filtered reads were aligned using the base bismark program, and duplicates were removed using deduplicate_bismark with default parameters. bismark_methylation_extractor was used to extract the methylated cytosines. deepTools (v3.5.1) [98] was used to calculate the methylation level of different gene categories.

### *MYB10* and *MYB114* identification and duplication analysis

The *MYB114* (accession number: MF489219) and *MYB10* (accession number: KT601121) pear coding sequences were downloaded from the NCBI database (https://www.ncbi.nlm.nih.gov/). The PAP1–PAP4 anthocyanin promoting MYB TFs in *A. thaliana* were downloaded from the TAIR database (https://www.arabidopsis.org/). The genome of YH1, and several other Rosaceae species were downloaded to identify *MYB10* and *MYB114* orthologs (Table S7, see online supplementary material). First, all MYB transcription factors were identified in each genome. Then BLAST software [77] was used to detect all candidate *MYB10* and *MYB114* genes with the *MYB10*, *MYB114*, and PAP1–PAP4 MYB TFs sequences as the query. MAFFT [92] was used to perform multiple sequence alignments. The alignment result file was used as input file, and a maximum-likelihood (ML) tree was constructed using IQ-TREE (v2.2.0) [99] with 1000 bootstrap replicates. The best substitution model was selected with the ModelFinder function. Finally, the MYB genes that clustered with the pear *MYB10* and *MYB114* genes or the *A. thaliana* PAP1–PAP4 MYB TFs were retained for further analysis (Fig. S9, see online supplementary material). The number of non-synonymous substitutions per synonymous site (Ka), synonymous substitutions per synonymous site (Ks), and the Ka/Ks ratios were calculated for these pairs using the NG method implemented in KaKs_Calculator (v3.0) [100].

### Weighted correlation network (WGCNA) analysis

Co-expression networks were constructed to identify gene modules with distinct expression patterns based on the TPM matrix using the WGCNA/R package [101]. RNA-seq data of 11 fruit skin samples (including D1, D2, D3, B1, B2, B3, 30 DAFB, 60 DAFB, 90 DAFB, 120 DAFB, and 150 DAFB) with three replicates were selected. Genes with a TPM value higher than one (in at least one sample) were selected for co-expression networks. KEGG enrichment analysis was performed with KOBAS (v3.0) [102].

### Transient transformation of pear fruits

For the transient transformation expression analysis, the full-length coding sequences of *MYB10–2* and *MYB114–2* were amplified from the pericarp cDNA of YH1 and inserted into the pCAMBIA1302 vector under the control of the 35S promoter. The recombinant plasmids were transformed into *Agrobacterium tumefaciens* strain GV3101 by the freeze–thaw method. *MYB10–2*-OE and *MYB114–2*-OE injected ten ‘Zaosu’ pears, and the blank control injected 10 fruits, respectively. The injected fruit were treated with continuous light for 5 days to observe the phenotype. The gene expression analysis by RT-qPCR was followed with previous descriptions [44]. Relative expression levels of each gene were calculated using the 2 − ΔΔCp algorithm. *PbrGAPDH* was used as reference genes for pear. The primer sequences were listed in Table S10 (see online supplementary material).

## Acknowledgements

This study was supported by the National Science Foundation of China (31820103012), National Key Research and Development Program (2022YFD1200503), Earmarked Fund for China Agriculture Research System (CARS-28), and Earmarked Fund for Jiangsu Agricultural Industry Technology System, China (JATS[2022]454). We thank the high-performance computing platforms of the Bioinformatics Center of Nanjing Agricultural University for supporting this project.

## Author contributions

J.W. designed this project and coordinated research activities; M.S. contributed to major data analysis; C.Y. performed the RNA-seq and WGCNA analysis. Q.S. and Y.H. collected and provided plant materials; G.C. and Z.X. performed the experiments. G.Y., and Y.L. provided valuable suggestions for analysis and manuscript writing. M.S. and J.W. interpreted data and contributed to writing the manuscript.

## Data availability

The raw reads generated in this study have been deposited in the CNCB genome sequence archive (GSA) with the accession number PRJCA019842 (https://ngdc.cncb.ac.cn/). The genome assembly and gene annotation data are available at database (http://pyrusgdb.sdau.edu.cn/).

## Conflict of interest statement

The authors declare no competing interests.

## Supplementary data

Supplementary data is available at *Horticulture Research* online.

## Supplementary Material

Web_Material_uhad201
