# Supplementary figures and images for: Telomere-to-telomere pear (Pyrus pyrifolia) reference genome reveals segmental and whole genome duplication driving genome evolution
Source: Hortic Res. 2023 Oct 12;10(11):uhad201. doi: 10.1093/hr/uhad201 (PMC10681005; doi:10.1093/hr/uhad201)

A

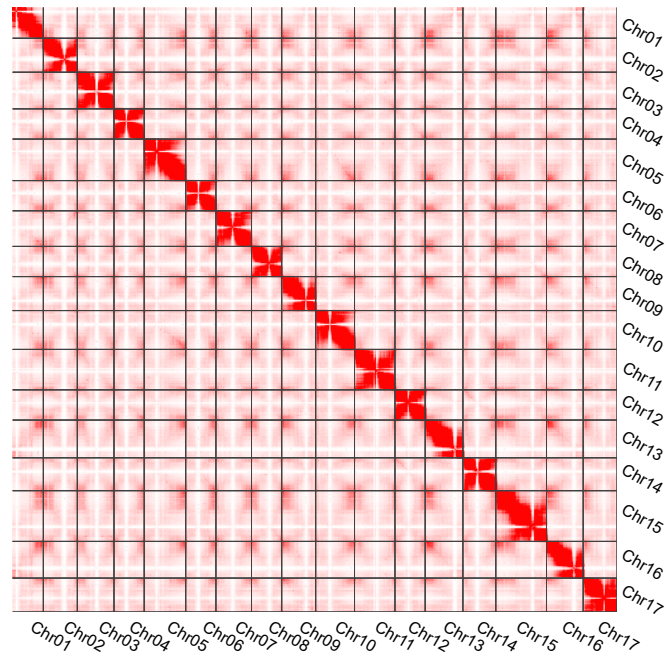

B

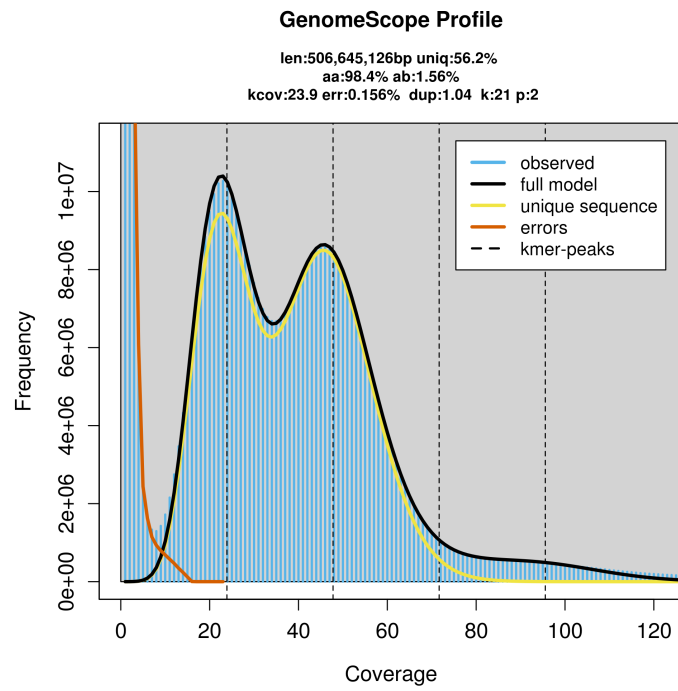

Supplement: Web_Material_uhad201 [file web_material_uhad201.zip › Figure S1.pdf]

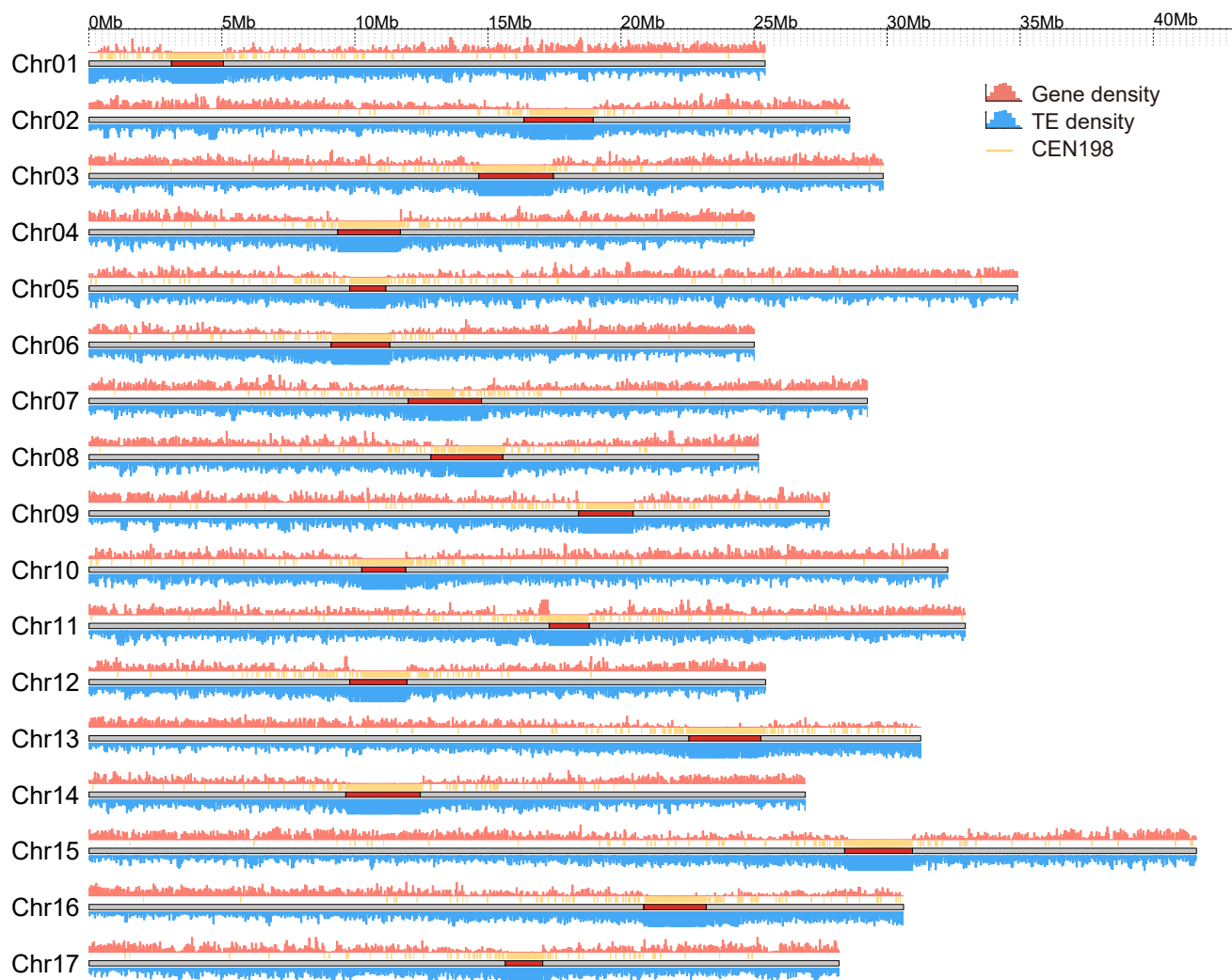

Supplement: Web_Material_uhad201 [file web_material_uhad201.zip › Figure S2.pdf]

# BUSCO Assessment Results

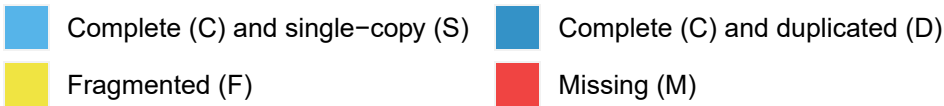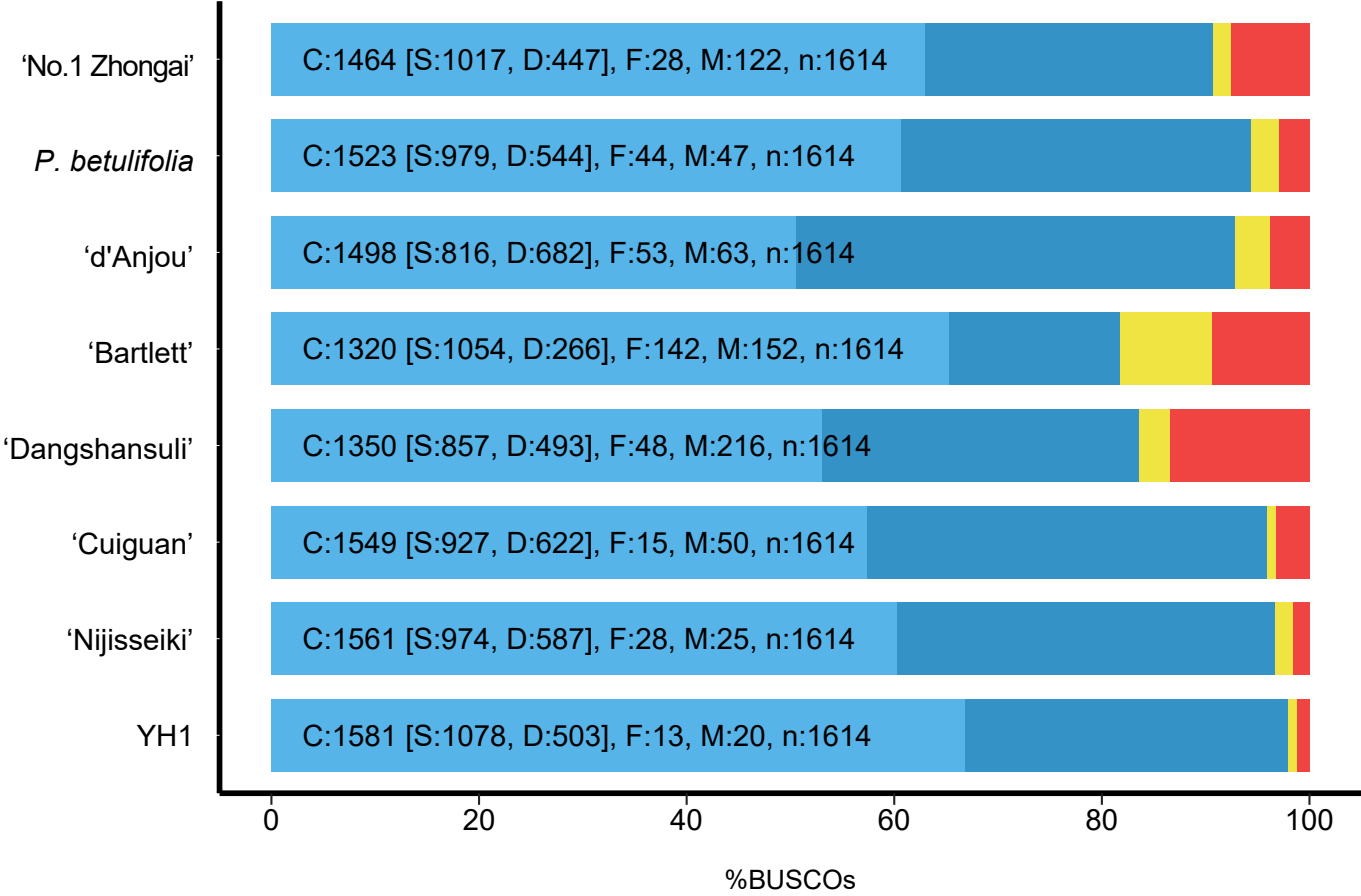

Supplement: Web_Material_uhad201 [file web_material_uhad201.zip › Figure S3.pdf]

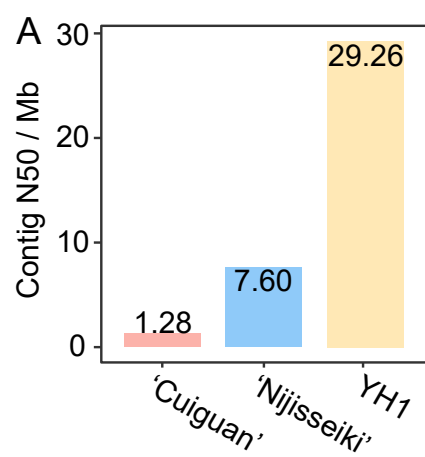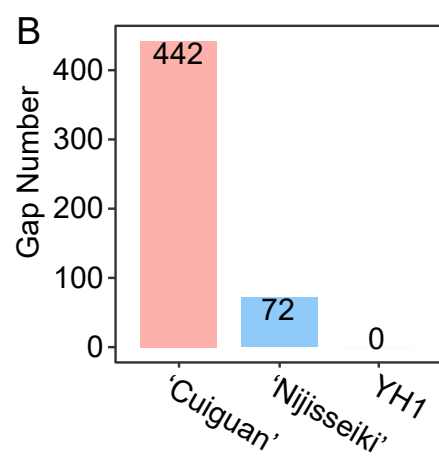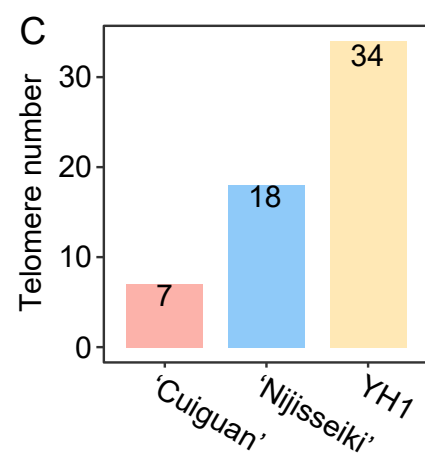

Supplement: Web_Material_uhad201 [file web_material_uhad201.zip › Figure S4.pdf]

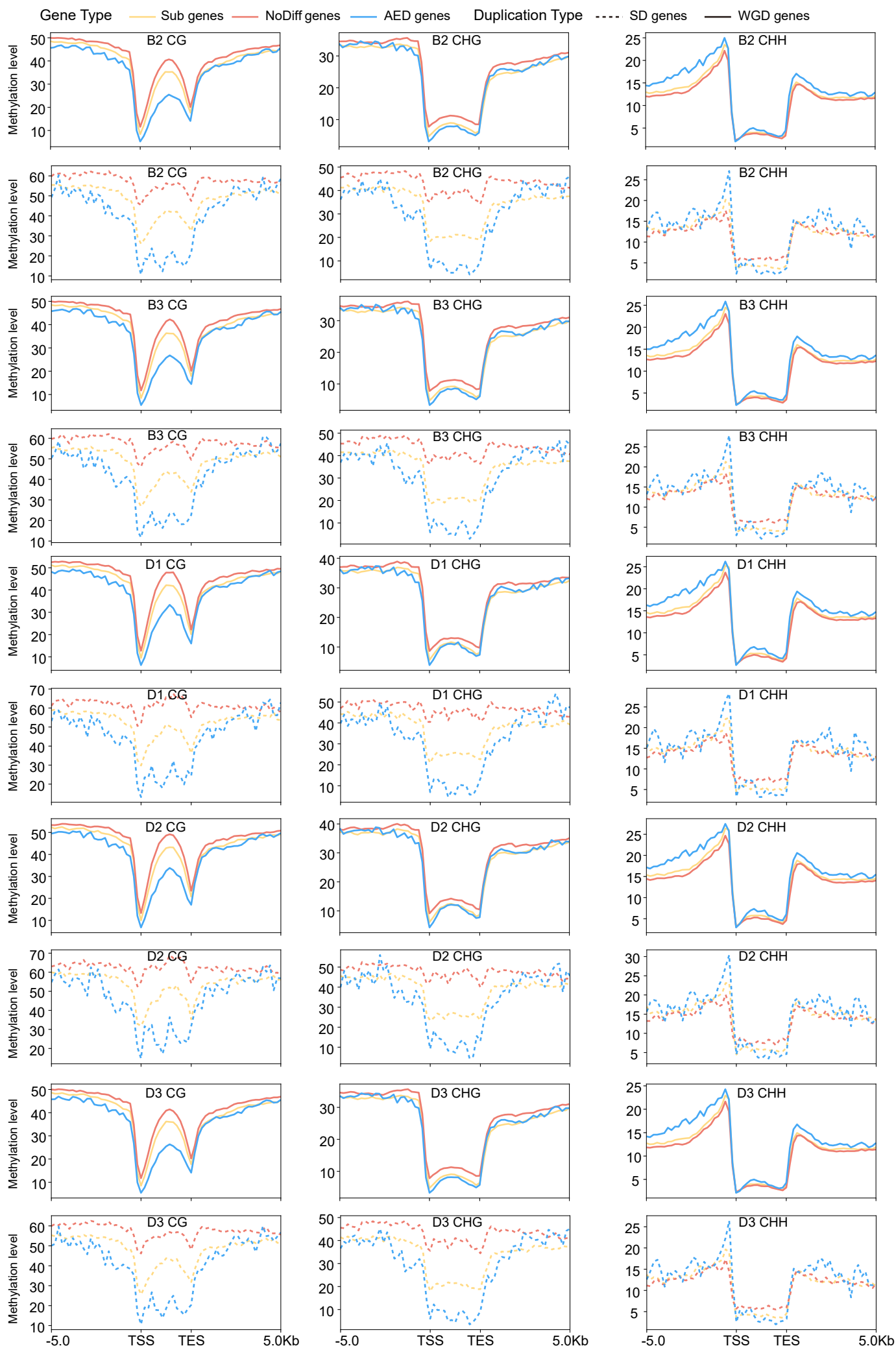

Supplement: Web_Material_uhad201 [file web_material_uhad201.zip › Figure S5.pdf]

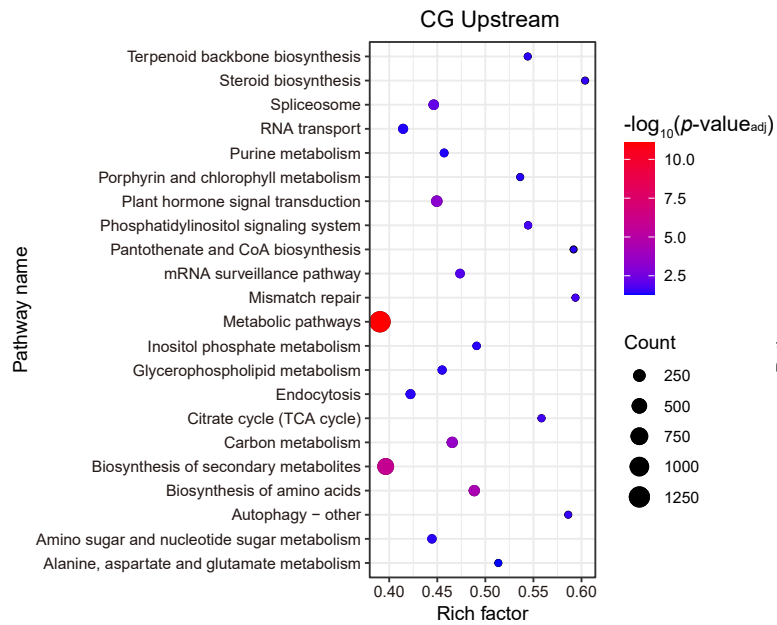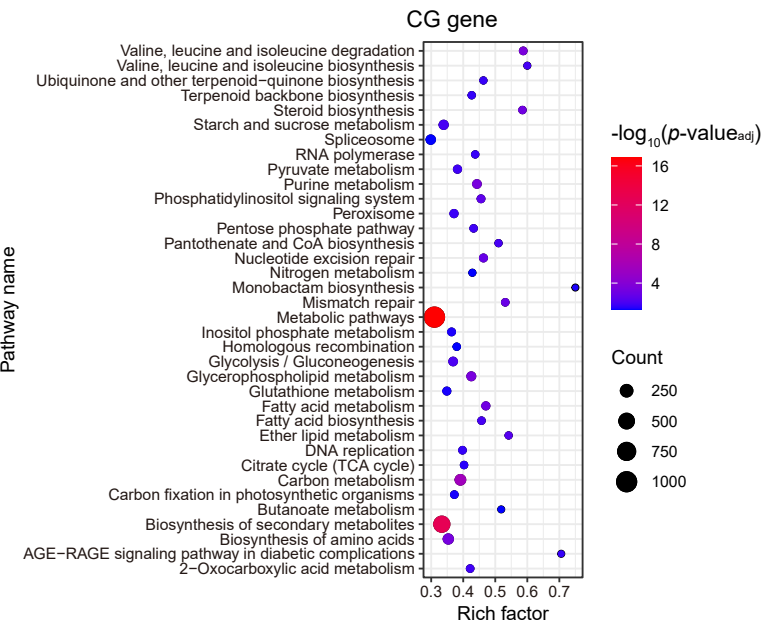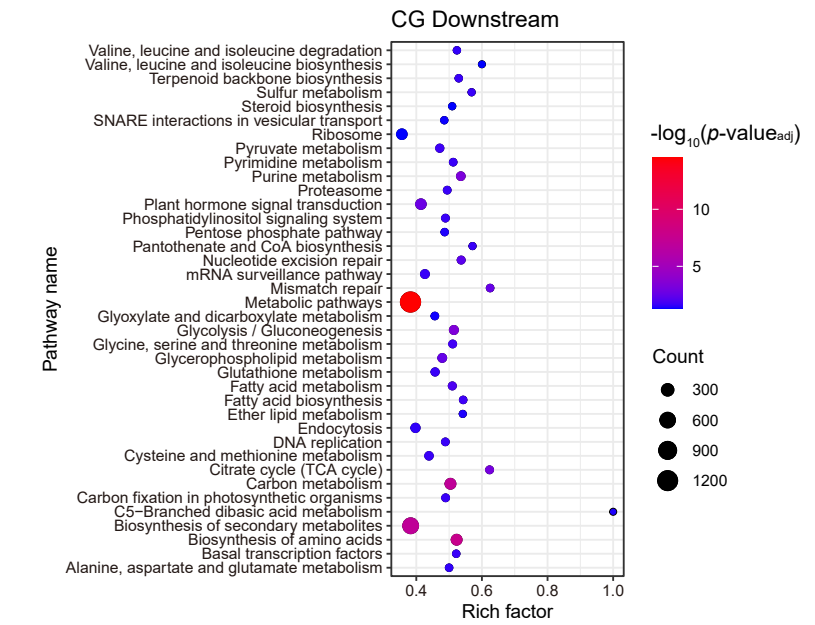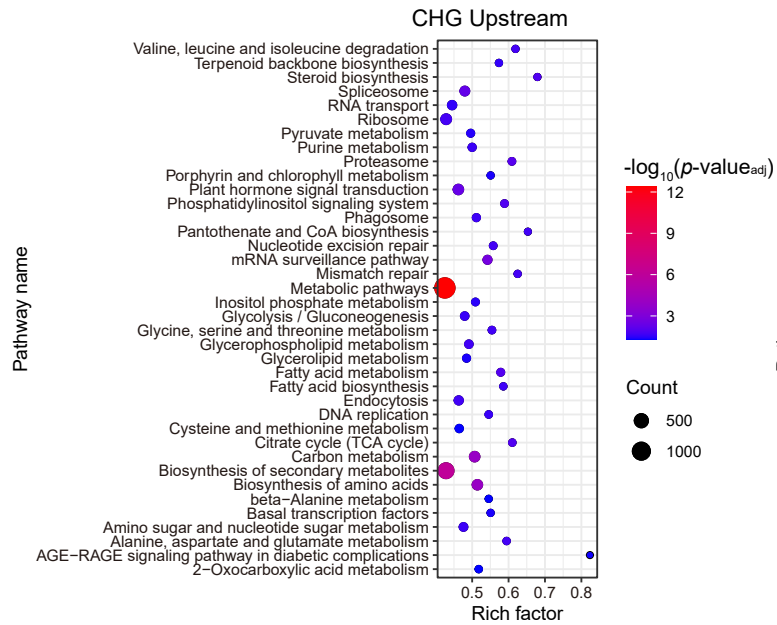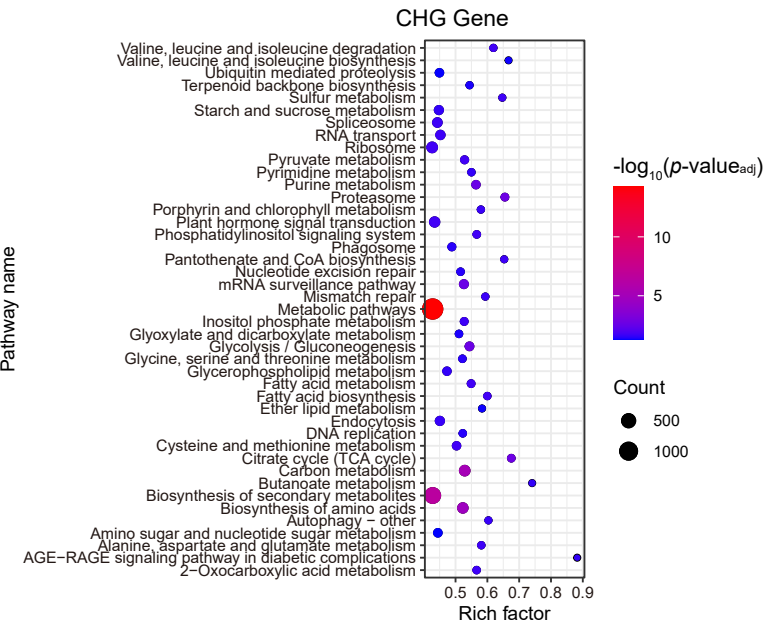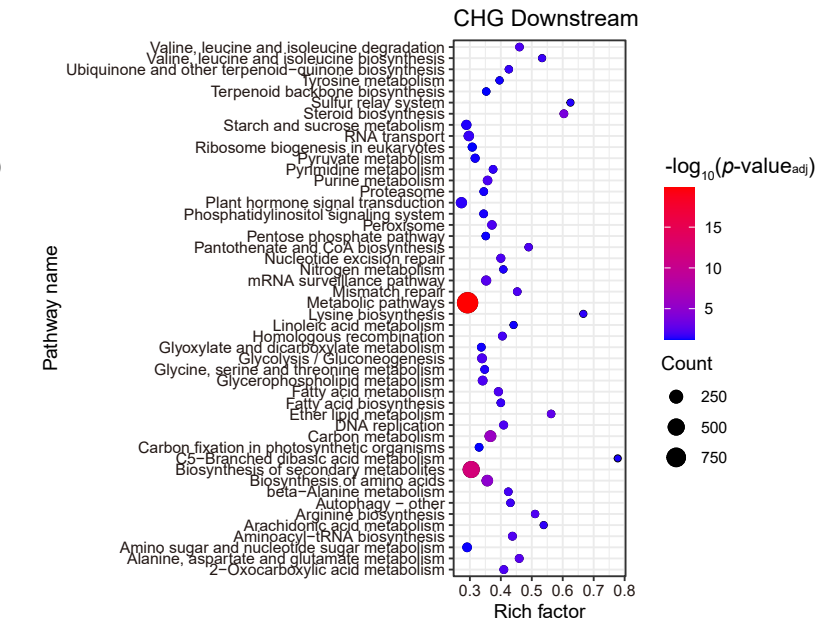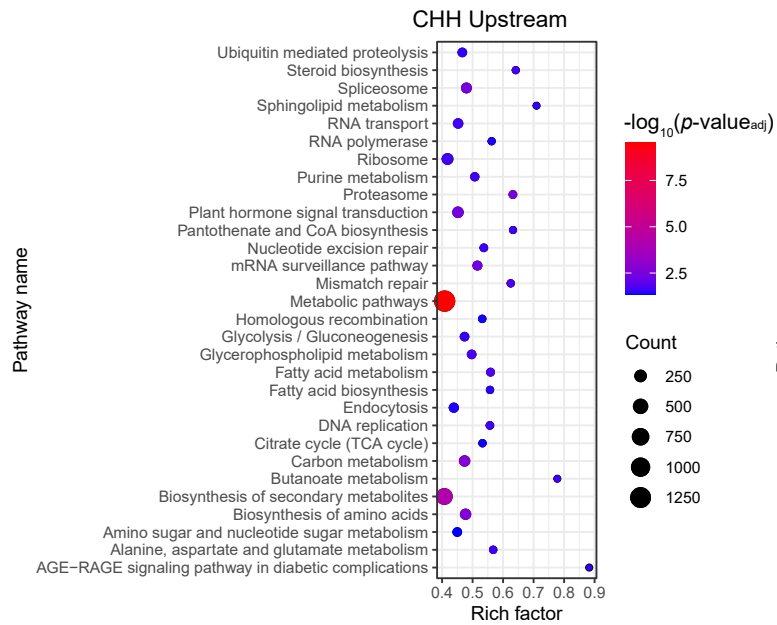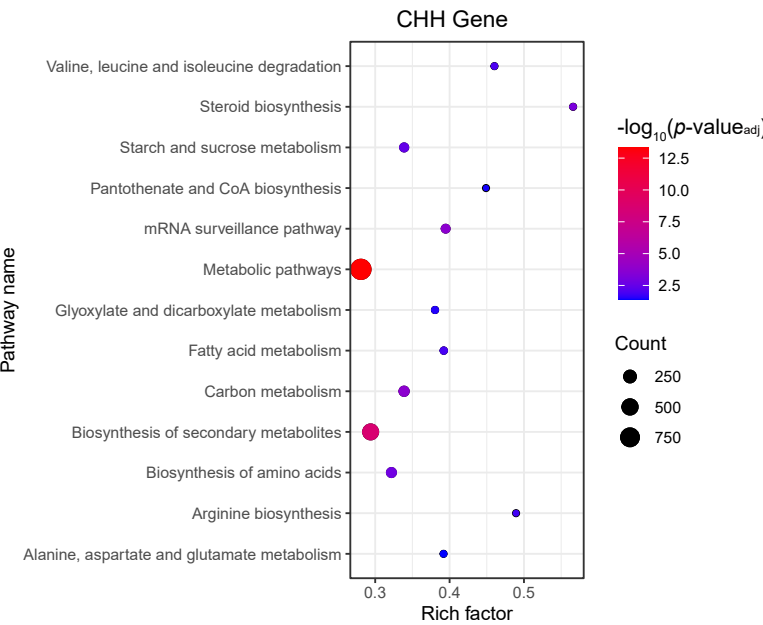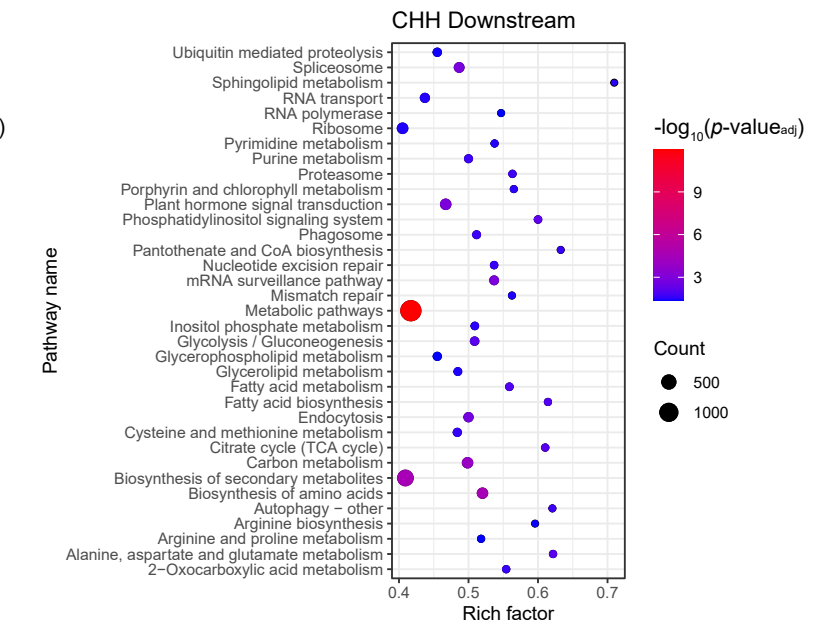

Supplement: Web_Material_uhad201 [file web_material_uhad201.zip › Figure S6.pdf]

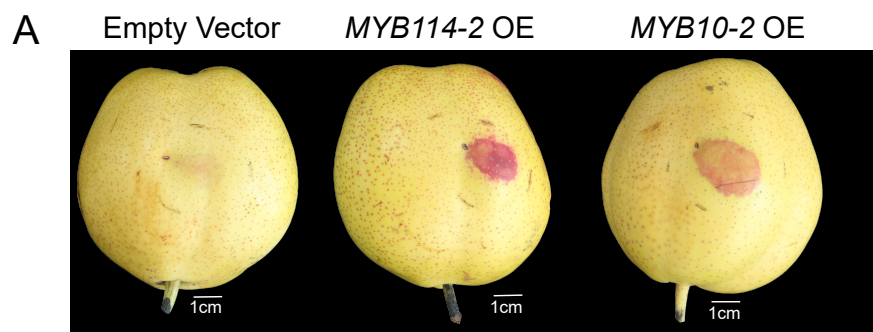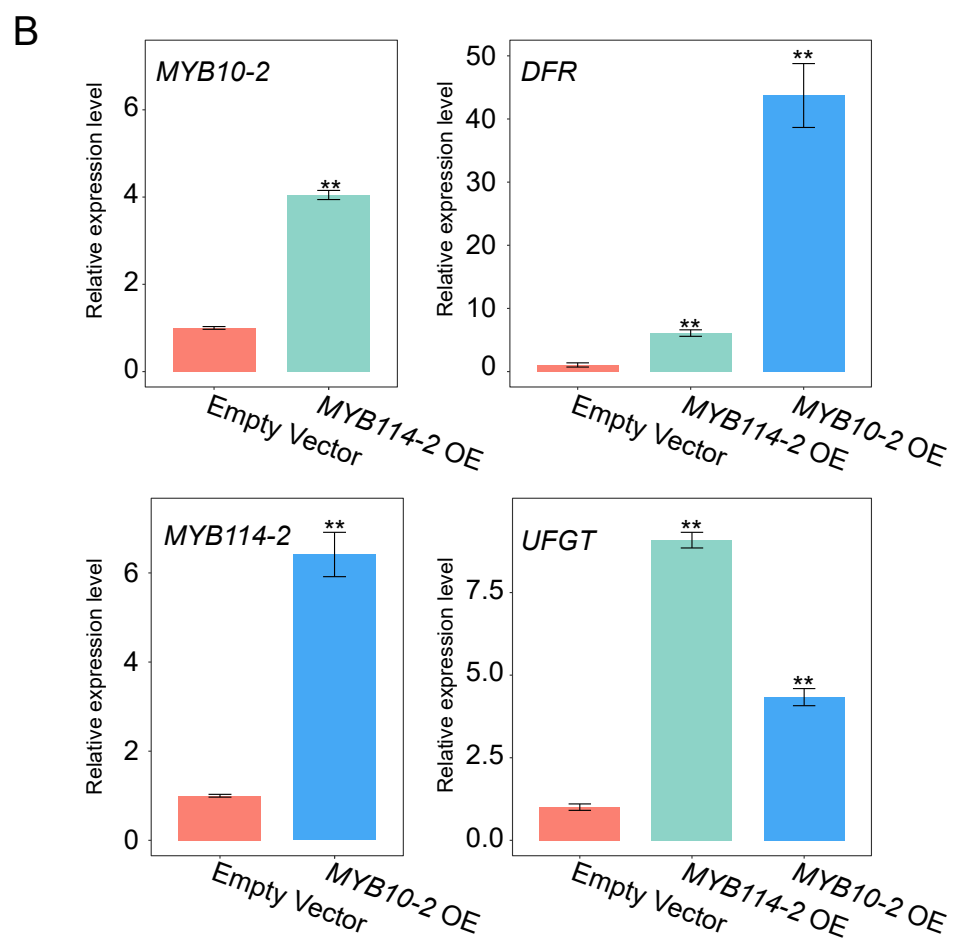

Supplement: Web_Material_uhad201 [file web_material_uhad201.zip › Figure S7.pdf]

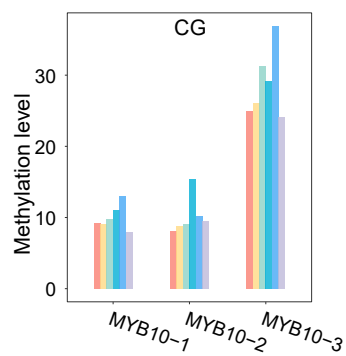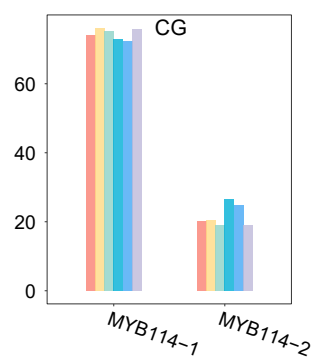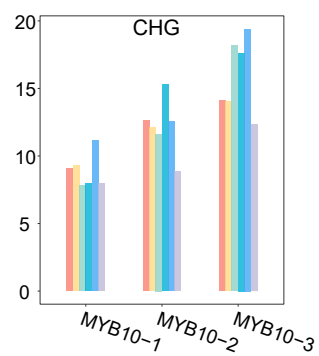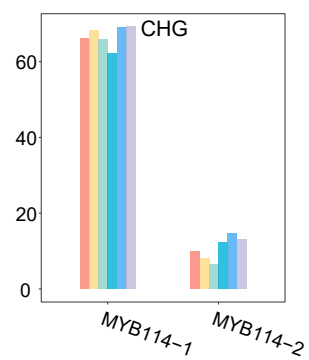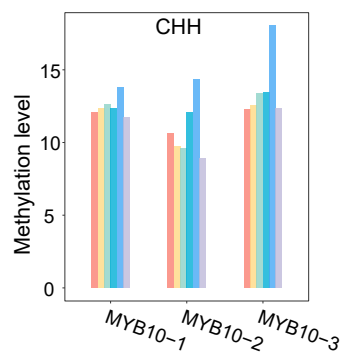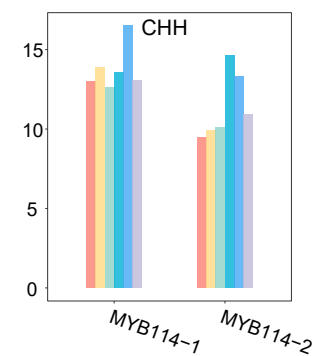

Sample

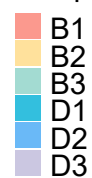

Supplement: Web_Material_uhad201 [file web_material_uhad201.zip › Figure S8.pdf]

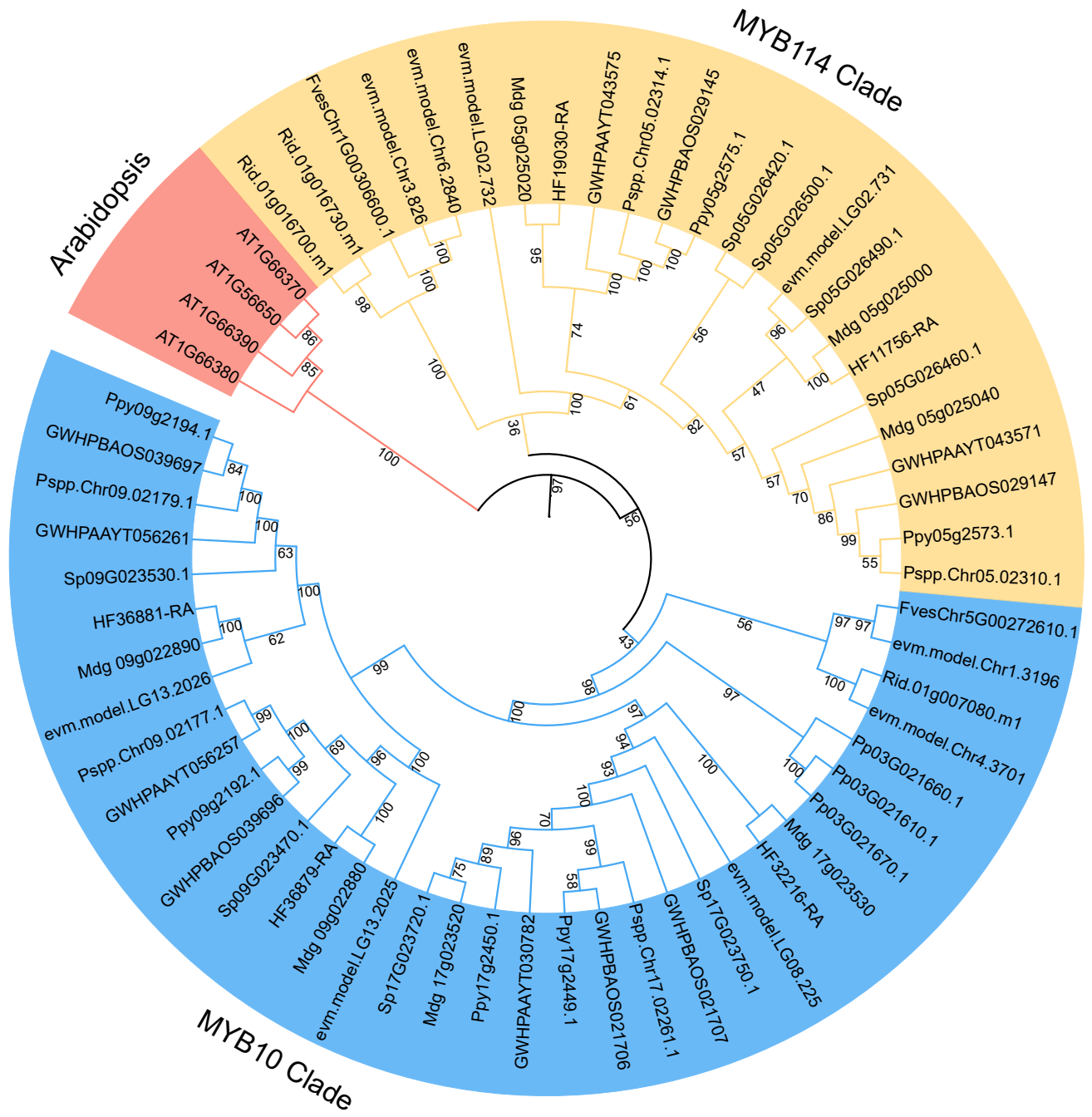

Supplement: Web_Material_uhad201 [file web_material_uhad201.zip › Figure S9.pdf]
